# Cross-correlation and multifractality analysis of the Chinese and American stock markets based on the MF-DCCA model

**DOI:** 10.1016/j.heliyon.2024.e36537

**Published:** 2024-08-22

**Authors:** Yijun Chen, Jun-hao Zhang, Lei Lu, Zi-miao Xie

**Affiliations:** a College of Finance, Guizhou University of Commerce, Avenida 26, 550014, Guiyang, PR China; b Faculty of Finance, City University of Macau, Avenida Xu Risheng Yin Gong, Taipa, 999078, Macao, PR China; c School of Psychological and Cognitive Sciences, Peking University, Yiheyuan Road, 100871, Beijing, PR China; d School of Finance, Shanghai Lida University, Cheting Road, 201319, Shanghai, PR China; e School of Business, Macau University of Science and Technology, Avenida Wailong, Taipa, 999078, Macao, PR China

**Keywords:** Chinese and American stock markets, Multifractal characteristics, Long-term memory, Nonlinearity, MF-DCCA model

## Abstract

**Objective:**

To compare the multifractal features and factors of the Chinese and American stock markets and their correlation, complexity and uncertainty.

**Methods:**

The paper analyzes the CSI 300 and S&P 500 indices from March 2018 to March 2023 using the MF-DCCA model and removes the long-term memory and nonlinear effects by random reshuffling and phase processing methods.

**Results:**

The paper shows that (1) CSI 300 and S&P 500 have multifractal features, with different long-term memory, complexity and irregularity at different scales; (2) The markets are fractal movements influenced by investors’ irrationality and expectations, not efficient markets; (3) Long-term memory and nonlinear effects cause the multifractal features. The paper offers a new perspective and method for the market investors and regulators.

## 1 Introduction

The stock market is an essential part of the national economic development and an indicator reflecting the national economic strength and competitiveness. As the two largest economies in the world, the performance of the Chinese and U.S. stock markets affects domestic investors and enterprises. It significantly impacts global financial stability and economic growth. The relationship between China and U.S. stock markets has undergone significant changes in recent years, affected by domestic and international political, economic, social, and other factors, such as the trade war, COVID-19, monetary policy, etc. On the one hand, the information transmission and capital flow between the two stock markets are increasingly rapid and frequent, showing a certain degree of linkage; on the other hand, there are also apparent differences and disunity between the two stock markets, reflecting the differences in economic structure and development stage between the two countries. Therefore, the relationship between China and U.S. stock markets is not a simple linear relationship but a complex and dynamic nonlinear relationship.

Exploring the correlation between China and U.S. stock markets and analyzing their characteristics, sources, changes, and influencing factors is of great theoretical significance and practical value for understanding the laws of operation of the two stock markets, evaluating the risks and returns of the two stock markets, and formulating reasonable investment strategies and regulatory policies. The cross-correlation between China's and U.S. stock markets is an important research topic involving the two stock markets' linkage, contagion, and risk spillover, which is significant to investors and regulators. Multifractality refers to the different fractal characteristics of a time series at different scales, reflecting the complexity, nonlinearity and non-stationarity. The multifractal detrended cross-correlation analysis method (MF-DCCA) (Podobnik, 2008, 2009, 2011) [[1], [2], [3]] is a method that can simultaneously analyze the cross-correlation and multifractality of two-time series, which can overcome the limitations of traditional linear correlation analysis methods and single fractal analysis methods. MF-DCCA is a combination of the multifractal detrended fluctuation analysis (MF-DFA) and detrended cross-correlation analysis (DCCA) methods, which can simultaneously consider the nonlinear and non-Gaussian characteristics of two-time series, as well as the differences and heterogeneity on different time scales and high-order statistical characteristics (Zhou, 2008) [4]. MF-DCCA has been widely used in the detection of cross-correlations between two financial series in some literature (Wang et al., 2010a, b; He and Chen, 2011a, b, c; Yuan et al., 2012) [[5], [6], [7], [8], [9], [10]].

This paper takes the two major stock indexes of China and the United States (CSI 300 and S&P 500) as the research objects, uses the MF-DCCA method to conduct cross-correlation analysis and multifractal analysis, and explores the time-varying, asymmetric, peak thickness and other characteristics of the cross-correlation and multifractal characteristics between China and U.S. stock markets from the perspectives of different time scales and high-order statistical characteristics. This paper aims to reveal the complex and dynamic correlation between China's and U.S. stock markets, evaluate the risks and returns of the two stock markets, and provide meaningful guidance and reference for investors and regulators. The research method of this paper is based on the cross-correlation analysis and multifractal analysis of the MF-DCCA method, which can simultaneously consider the nonlinear and non-Gaussian characteristics of two-time series, as well as the differences and heterogeneity on different time scales and high-order statistical characteristics. This paper has the following mainly marginal contributions: First, from the perspective of different time scales, it analyzes the changes of cross-correlation and multifractal characteristics between China and U.S. stock markets over time and reveals that the two stock markets have different degrees and directions of linkage effect at different time scales. Second, from the perspective of different high-order statistical characteristics (such as skewness and kurtosis), it analyzes the cross-correlation and multifractal characteristics between China and U.S. stock markets under positive and negative fluctuations and reveals that the two stock markets have different degrees and directions of asymmetric effect on different high-order statistical characteristics, and reveals that the two stock markets have different degrees and directions of tail thickness effect on different high-order statistical characteristics. Third, from the theoretical and practical perspectives, it provides crucial guidance and reference for investors to grasp the correlation and multifractal characteristics between China and U.S. stock markets, evaluate the risks and returns of the two stock markets, and formulate reasonable investment strategies and regulatory policies.

In light of these considerations, the present study is dedicated to exploring the extant research on the dynamic interactions between these significant markets, with a systematic effort to identify gaps in current understanding. Utilizing the multifractal detrended cross-correlation analysis (MF-DCCA) method, this investigation aims to unveil detailed insights into the multifractal features, driving factors, and their implications for investors and policy-makers. Such an endeavor aspires to contribute to a more sophisticated understanding of global financial stability. The subsequent sections will engage in an in-depth analysis of existing literature, scrutinizing the advancements in research concerning the cross-correlation and multifractal characteristics of the Chinese and U.S. stock markets. Additionally, this examination will highlight how these scholarly contributions enhance our comprehension of the dynamics within global financial markets.

## 2 Literature review and hypothesis

The cross-correlation and multifractality between Chinese and U.S. stock markets are important and complex issues in finance. Cross-correlation reflects the degree of mutual association between two time series, which can reveal the interaction and information transmission mechanism between two markets and has important guiding significance for investors to grasp market changes and adjust investment strategies; multifractality reflects whether the statistical characteristics of a time series are consistent at different time scales or under different fluctuations, which can reveal the heterogeneity, nonlinearity, non-stationarity and other characteristics within a market, and has essential significance for investors to evaluate market risks and returns. Therefore, an in-depth exploration of the cross-correlation and multifractality between Chinese and U.S. stock markets can enhance investors' and regulators' understanding of the essential laws and dynamic evolution process and provide investors and regulators with more effective investment decisions and regulatory policies.

At present, many scholars at home and abroad have studied the cross-correlation and multifractality between Chinese and U.S. stock markets. According to different research methods, these studies can be roughly divided into the following categories.

(1) Studies based on linear vs. nonlinear correlation analysis methods. Research in this domain typically oscillates between employing linear correlation analysis methods, such as cointegration analysis, vector autoregressive models, and Granger causality tests, and nonlinear correlation analysis methods, including mutual information, transfer entropy, and interdependence approaches. Linear methods have been instrumental in uncovering long-term equilibrium relationships, dynamic interactions, and causal relationships between the Chinese and U.S. stock markets. These approaches, rooted in solid theoretical foundations, are adept at parameter estimation but are constrained by assumptions of linear correlation, stationarity, or cointegration, which may not hold in the inherently nonlinear and nonstationary nature of financial time series. They tend to provide an average or overall correlation perspective, potentially overlooking the nuances of correlation at varying time scales or under different market conditions. For instance, Geng Yanqiu et al. (2017) [11], Chen Liu (2013) [12], and Ai Yongfang et al. (2009) [13] identified long-term equilibrium relationships and significant interactions between the Chinese and U.S. stock markets using these linear methods.

Conversely, nonlinear correlation analysis methods excel at detecting nonlinear interactions and information transmission nuances between these markets. These methods offer robustness against the variegated probability distributions and fractal dimensions characteristic of financial time series, although they too have limitations. Primarily, these approaches presuppose identical probability distributions or fractal dimensions across time series, a condition seldom met in financial datasets. Moreover, while capable of capturing the essence of overall or average information transmission, they fall short of delineating the disparities in information transmission across different time scales or during varied market fluctuations. Notably, Gai Wei and Hu Jie (2021) [14] and Wang Xudong (2019) [15] utilized these nonlinear methods to reveal the intricate dynamics of information flow and interaction patterns between the Chinese and U.S. stock markets, particularly highlighting the intensified information transmission during market downturns and the predominance of the U.S. market's influence on its Chinese counterpart.

This juxtaposition of linear and nonlinear methodologies underscores the complexity and multifaceted nature of analyzing correlations between the Chinese and U.S. stock markets. Each approach contributes valuable insights into the intricate web of global financial interactions, yet also underscores the inherent limitations and the necessity for a more integrative and nuanced analytical framework to fully comprehend the dynamism of stock market correlations.

(2) Studies based on applications of MF-DCCA in market analysis. This type of research mainly uses the MF-DCCA method or its improved method to explore the cross-correlation and multifractal characteristics between different markets. Further, it analyzes its time-varying characteristics, influencing factors and predictive ability. This type of method has strong nonlinear, nonstationary and non-Gaussian processing ability and good adaptability, but also has some limitations: first, they need to assume that the two-time series have the same trend function or the same detrending method and other characteristics, while in fact, financial time series often have different trend functions or different detrending methods and other characteristics; second, they can only reflect the overall or average multifractal characteristics between the two-time series, and cannot reflect the differences in multifractal characteristics between the two-time series at different time scales or under different fluctuations. For example, Ruan et al. (2018) [16] used MF-DCCA method to find that the cross-correlation and multifractal characteristics between Hong Kong stock market and mainland stock market have significant differences in different volatility-constrained intervals and are affected by essential events in financial market; Li Shuping et al. (2020) [17] used MF-DCCA method based on wavelet transform to find that the cross-correlation and multifractal characteristics between stock market, currency market and foreign exchange market have significant differences at different time scales, and have time-varying characteristics; Huang Jianbai et al. (2013) [18] used MF-DCCA method based on empirical mode decomposition to find that there is a relationship between the quantity and price of metal futures, and it has predictive ability.
(3) Effects of global events on market correlation. In recent years, with significant events such as Sino-U.S. trade frictions and the COVID-19 pandemic, the cross-correlation and multifractal characteristics between Chinese and U.S. stock markets have also changed. Some scholars have used more advanced methods and updated data to conduct updated research on the cross-correlation and multifractal characteristics between Chinese and U.S. stock markets. These methods have strong time-varying processing ability and sound sensitivity but also have some limitations: first, they need to assume that the two-time series have the same structural breakpoints or the same state space model and other characteristics, while in fact, financial time series often have different structural breakpoints or different state space models and other characteristics; second, they can only reflect the overall or average time-varying characteristics between the two-time series, and cannot reflect the differences in time-varying characteristics between the two-time series in different periods or different states. For example, Zhang Hongmei et al. (2023) [19] used the EMD-MF-DCCA method to find that in the Shanghai and Shenzhen stock markets, the two markets show long-range correlation in large fluctuations and persistent characteristics in small fluctuations, and have an asymmetric multifractal relationship; Zhou Yinggang et al. (2022) [20] derived a spatial autoregressive (SAR) model from the static general equilibrium model, and the results showed that the impact of bilateral exchange rate changes between China and the United States on the stock markets of the two countries was more significant during the trade frictions than before; Li Helong et al. (2019) [21] used the ensemble empirical mode decomposition (EEMD) method to find that there are significant differences in the linkage between Chinese and U.S. stock markets in short, medium and long cycles; Duan Daiwei et al. (2022) [22] based on the DCC-MGARCH and TVP-SP-VAR two time-varying coefficient models, found that the linkage between Chinese and U.S. stock markets has significant time-varying characteristics, and is affected by globalization, RMB internationalization and the COVID-19 pandemic.

In summary, many scholars at home and abroad have conducted in-depth studies on the cross-correlation and multifractality between Chinese and U.S. stock markets. However, there are still some problems and deficiencies. First, regarding research methods, most studies only focus on the static cross-correlation or overall cross-correlation between Chinese and U.S. stock markets and ignore the dynamic or local cross-correlation [23]. Dynamic cross-correlation reflects the changes of cross-correlation between two-time series over time, which can reveal the time-varying characteristics of interaction and information transmission between the two time series; local cross-correlation reflects the differences of cross-correlation between two time series at different time scales or under different fluctuations, which can reveal the heterogeneity, nonlinearity, non-stationarity and other characteristics of the two time series. These characteristics have important guiding significance for investors to grasp market changes and adjust investment strategies. Second, regarding research content, most studies only focus on the linear or nonlinear cross-correlation between Chinese and U.S. stock markets and ignore the non-Gaussian cross-correlation. Non-Gaussian cross-correlation reflects the high-order statistical characteristics between two-time series. This can reveal the asymmetry, tail thickness, and other characteristics between the two time series and have important significance for investors in evaluating market risks and returns. Therefore, this paper believes that conducting a more in-depth study on the dynamic and non-Gaussian cross-correlation between Chinese and U.S. stock markets will help obtain more comprehensive and accurate research results.

Drawing from the identified research gaps, particularly the insufficient exploration of non-linear correlations and the impact of global events, this paper proposes the following research hypotheses.

Hypothesis 1 There is nonlinear and non-Gaussian cross-correlation between Chinese and U.S. stock markets, which is manifested as asymmetry, sharp-peaked and thick-tailed, and has significant time-varying characteristics;

Hypothesis 2 Chinese and U.S. stock markets do not conform to the efficient market theory hypothesis but rather conform to the fractal market theory hypothesis;

Hypothesis 3 The long memory effect and nonlinear effect between Chinese and U.S. stock markets are the main reasons for their multifractal characteristics.

In order to further explore the cross-correlation and multifractal characteristics between Chinese and U.S. stock markets, this paper will use the MF-DCCA method and its improved methods to analyze the dynamic cross-correlation and non-Gaussian cross-correlation between Chinese and U.S. stock markets. MF-DCCA is a cross-correlation analysis method proposed by Podobnik et al. (2008, 2009, 2011) [[1], [2], [3]] based on sliding window technology and detrending technology, which can simultaneously consider the nonlinear, nonstationary and non-Gaussian characteristics of two-time series, as well as the differences in correlation at different time scales. The strength of this method lies in its ability to analyze the cross-correlation and multifractal characteristics of two time series without the need for assuming that they share the same fractal dimension or probability distribution, thereby overcoming the limitations of traditional linear correlation analysis methods and singular fractal analysis approaches.

## 3 Research design

With the hypotheses clearly delineated, this paper proceeds to describe the methodology employed in the study. This section explicates the research design, data collection, and analytical techniques, ensuring a rigorous approach to testing the propositions.

### 3.1 Selecting sample

In the Sino-US securities market, CSI 300 and S&P 500 are the main parameters reflecting the overall performance of the two countries’ stock market Therefore, selecting the CSI 300 and S&P 500 indexes obtained based on daily transaction data as the research objects to examine the volatility characteristics of China-US capital and financial markets is representative. In order to eliminate the impact of price level differences on the calculation of return rates and, at the same time, enhance the stationarity and normality of the return rate sequence, the daily transaction index data sequence is converted into a logarithmic return rate form, which is calculated using Equation (1):

(1) $$r_{t}=\ln\left(p_{t+1}/p_{t}\right)\;t=1,2,\cdots ,N\text{,}$$

In the formula, $p_{t}$ is the stock market index value corresponding to the t trading day, and $r_{t}$ is the return rate at the time t.

### 3.2 Methodology

In order to explore the cross-correlation and multifractal characteristics between Chinese and U.S. stock markets and to analyze their time-varying, asymmetric, tail thickness and other characteristics from the two perspectives of different time scales and high-order statistical characteristics, this paper uses the MF-DCCA method proposed by Podobnik et al. (2008) [1] and improved by Zhou et al. (2008, 2019) [24,25] to conduct cross-correlation analysis and multifractal analysis on the two-time series.

(1) Cross-correlation test. This paper uses the cross-correlation statistic constructed by Podobnik et al. (2009) [2] to test whether there is a cross-correlation between any two-time series of equal length. For two nonstationary time series $\left\{x_{t}\right\}$、 $\left\{y_{t}\right\}$, t=1,2,⋯,N, the cross-correlation function $c_{i}$ is demonstrated in Equation (2):

(2) $$c_{i}=\frac{\sum_{k=i+1}^{N}x_{k}y_{k-i}}{\sqrt{\sum_{k=1}^{N}x_{k}^{2}\sum_{k=1}^{N}y_{k}^{2}}}$$

The cross-correlation statistic $Q_{cc}\left(m\right)$ is demonstrated in Equation (3):

(3) $$Q_{cc}\left(m\right)=N^{2}\sum_{i=1}^{m}\frac{c_{i}^{2}}{N-i}$$

The null hypothesis of the cross-correlation statistic $Q_{cc}\left(m\right)$ test is: as the degree of freedom m changes, the value of $Q_{cc}\left(m\right)$ is approximately equal to the chi-square distribution $\chi^{2}\left(m\right)$, then the two-time series do not have a cross-correlation relationship; otherwise, there is a cross-correlation relationship. The degree of freedom is from 1 to 1000.

(2) DCCA Correlation Coefficient [19]。

The calculation formula for the DCCA correlation coefficient is shown in Equation (4):

(4) $$\rho_{DCCA}=\frac{F_{DCCA}^{2}\left(s\right)}{F_{DFA}\left(s\right)F_{DFA}^{\prime }\left(s\right)}$$

In which, $F_{DCCA}^{2}\left(s\right)$ is the cross-covariance function of the detrended fluctuations of two columns of time series, $F_{DFA}\left(s\right)$ and $F_{DFA}^{\prime }\left(s\right)$ are the variances of the fluctuations of the time series after detrending, respectively. Unlike the traditional Pearson correlation coefficient, the DCCA correlation coefficient does not require a strictly stationary time series to be unbiasedly estimated, and the DCCA coefficient will get different values for different time scales [17].

(3) Multifractal analysis. For two nonstationary time series $\left\{x_{t}\right\}$, $\left\{y_{t}\right\}$, t=1,2,⋯,N, the MF-DCCA model is applied to study the multifractal characteristics of China-US stock markets. The main steps are as follows [[26], [27], [28], [29]]:

**Step 1:** To examine the cumulative deviations of original time series $\left\{x_{t}\right\}$, $\left\{y_{t}\right\}$, t=1,2,⋯,N, new time series X(i), Y(i) are constructed as shown in Equation (5). Here, $\bar{x}$ and $\bar{y}$ represent the mean values of the $\left\{x_{t}\right\}$ and $\left\{y_{t}\right\}$ series, respectively, as described in Equation (6). These new cumulative series are then used to further the analysis, as described in Equation (5).

(5) $$X\left(i\right)=\sum_{k=1}^{i}\left(x_{k}-\bar{x}\right),Y\left(i\right)=\sum_{k=1}^{i}\left(y_{k}-\bar{y}\right)\text{,}$$

(6) $$\bar{x}=\frac{1}{N}\sum_{t=1}^{N}x_{t},\bar{y}=\frac{1}{N}\sum_{t=1}^{N}y_{t}\;i=1,2,\cdots ,N\text{,}$$

**Step 2:** Divide the two time series X(i), Y(i) into $N_{s}$ non-overlapping equal-length subintervals v of length s, where $N_{s}=\left[N/s\right]$ (i.e., take an integer). Since the lengths of the sequences X(i), Y(i) are not always an integer multiple of the time length s, a small part of the contour sequence data is not utilized. In order to make full use of this small end sequence, the reverse repetition of this division operation is taken for X(i), Y(i), thus obtaining a total of 2 $N_{s}$ equal-length subintervals.

**Step 3:** Use the least squares method to fit the data on each subinterval v ($v=1,2,\cdots ,2N_{s}$) to obtain the local trend function $p_{v}\left(i\right)$ of the interval. After filtering out the local trend in the subinterval v (the difference between the original sequence and the fitted value), the time series is denoted as $X_{s}\left(i\right)$、 $Y_{s}\left(i\right)$. The time series is detrended using the fitted polynomial as described in Equation (7):

(7) $$X_{s}\left(i\right)=X\left(i\right)-p_{v}\left(i\right),Y_{s}\left(i\right)=Y\left(i\right)-p_{v}\left(i\right),i=1,2,\cdots ,2N_{s}\text{,}$$

In the formula, $p_{v}\left(i\right)$ is the fitted polynomial of the v-th interval. The fitting polynomial $p_{v}\left(i\right)$ can adopt linear, quadratic, cubic, or higher-order m polynomials.

**Step 4:** The variance of each subinterval v after detrending is calculated using the formula provided in Equation (8):

$$F^{2}\left(v,s\right)=\frac{1}{s}\sum_{i=1}^{s}\left\{X\left[\left(v-1\right)s+i\right]-x_{v}\left(i\right)\right\}\times \left\{Y\left[\left(v-1\right)s+i\right]-y_{v}\left(i\right)\right\},v=1,2,\cdots ,N_{s}\text{,}$$

(8) $$F^{2}\left(v,s\right)=\frac{1}{s}\sum_{i=1}^{s}\left\{X\left[N-\left(v-N_{s}\right)s+i\right]-x_{v}\left(i\right)\right\}\times \left\{Y\left[N-\left(v-N_{s}\right)s+i\right]-y_{v}\left(i\right)\right\},v=N_{s}+1,N_{s}+2,\cdots ,2N_{s}\text{,}$$

**Step 5:** The q-order MF-DCCA fluctuation function of the time series is calculated using the method outlined in Equation (9):

(9) $$F_{q}\left(s\right)=\left\{ \begin{array}{c} {\left[\frac{1}{2N_{s}}\sum_{v=1}^{2N_{s}}{\left(F^{2}\left(v,s\right)\right)}^{q/2}\right]}^{1/q},q\neq 0;\\ \exp\left[\frac{1}{4N_{s}}\sum_{v=1}^{2N_{s}}\ln\left(F^{2}\left(v,s\right)\right)\right],q=0;\; \end{array}\right.$$

**Step 6:** For different values of q, analyze the relationship $F_{q}\left(s\right)∼s^{h_{xy}\left(q\right)}$ between the fluctuation function $F_{q}\left(s\right)$ and the observation scale *s.* For each *s*, find the corresponding $F_{q}\left(s\right)$, and use the least squares method to fit $\ln\left(F_{q}\left(s\right)\right)∼\ln\left(s\right)$, and the obtained slope is the scale index, called the q-order generalized Hurst index $h_{xy}\left(q\right)$. If the size of $h_{xy}\left(q\right)$ is independent of the parameter q, then the sequences $\left\{x_{t}\right\}$, $\left\{y_{t}\right\}$ have monofractal characteristics; otherwise, when h(q) changes with the parameter q, the two-time series have multifractal characteristics.

The generalized Hurst index $h_{xy}\left(q\right)$ characterizes the correlation characteristics of the two-time series. When the scale index $h_{xy}\left(q\right)=0.5$, it means that the two-time series are uncorrelated and are independent random processes. When $0<h_{xy}\left(q\right)<0.5$, it indicates that the two time series have anti-persistence characteristics. When $0.5<h_{xy}\left(q\right)<1$, it indicates that the two time series have long-range correlation (fractal) or persistence. If there is an upward (or downward) trend before time t, it implies that there is generally an upward (or downward) trend after time t. Past events affect the future, and there is a correlation between events across time scales. Its trend enhancement behaviour depends on the degree of $h_{xy}\left(q\right)>0.5$.

According to the multifractal theory [16], the relationship between the Hurst index $h_{xy}\left(q\right)$ and the Renyi index $\tau_{xy}\left(q\right)$ is given by Equation (10):

(10) $$\tau_{xy}\left(q\right)=qh_{xy}\left(q\right)-1\text{,}$$

Thus, the Holder index $\alpha_{xy}$ and the multifractal spectrum $f_{xy}\left(\alpha \right)$ can be obtained through Legendre transform [30], as described in Equation (11):

(11) $$\left\{ \begin{array}{c} \alpha_{xy}=\frac{d\tau_{xy}\left(q\right)}{dq}=h_{xy}\left(q\right)+{\text{qh}}_{xy}^{\prime }\left(q\right)\text{,}\\ f_{xy}\left(\alpha \right)=q\alpha_{xy}\left(q\right)-\tau_{xy}\left(q\right)=q\left[\alpha_{xy}\left(q\right)-h_{xy}\left(q\right)\right]+1\text{,} \end{array}\right.$$

In the formula, $\alpha_{xy}$ is the singularity intensity, which is used to describe the different singularity degrees of each interval in a complex system. Let $\alpha_{0}$ represent the value of $f_{xy}\left(\alpha \right)$ when $\alpha_{xy}$ takes the maximum value, $\alpha_{\min}$ and $\alpha_{\max}$ represent the minimum and maximum values of $\alpha_{xy}$, respectively. $\Delta \alpha_{xy}=\alpha_{\max}-\alpha_{\min}$ represents the width of the fractal spectrum, which represents the unevenness of the probability change; that is, the larger the $\Delta \alpha_{xy}$, the more uneven the time series distribution, and the more pronounced the multifractal characteristics. $f_{xy}\left(\alpha \right)$ is the multifractal spectrum, and its value reflects the fractal dimension of the singularity index α. If the time series under study has monofractal characteristics, then the function $f_{xy}\left(\alpha \right)$ is a constant; if the time series under study has multifractal characteristics, then the multifractal spectrum $f_{xy}\left(\alpha \right)$ graph is a single-peak bell-shaped curve.

## 4 Empirical results analysis

Upon applying the outlined methodological framework, this paper presents the findings. This section details the results of the analysis, providing empirical evidence to either support the initial hypotheses.

### 4.1 Descriptive statistics

The sample data selected in this paper spans from March 15, 2018, to March 15, 2023. The two countries’ days off due to national holidays differed during this period. The Chinese stock market traded for 1217 days, and the U.S. stock market traded for 1259 days. A total of 1217 sample data were selected, and after converting them into logarithmic returns, 1216 sample observations were formed. Data source: Investing.com website (https://www.investing.com/).

**Fig. 1:**
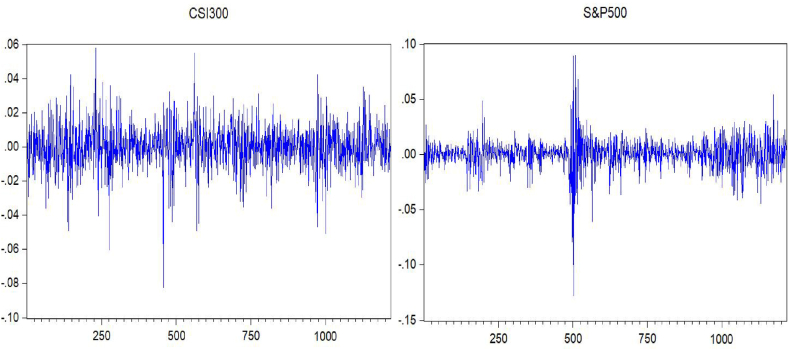
CSI 300 and S&P 500 price earnings ratio.

It can be seen from Fig. 1 that in the five years from March 2018 to March 2023, the yield of CSI 300 fluctuated wildly, indicating that CSI 300 was affected by multiple factors during this period, including the Sino-US trade war, the COVID-19 pandemic and China's economic policy adjustments. The S&P 500 was relatively stable during the same period, with only a significant fluctuation at around 500 when the COVID-19 pandemic peaked in the United States. In contrast, the CSI 300 and S&P 500 yield trends are somewhat correlated but must be more consistent. The two indices reflect the conditions of their respective markets but also have their characteristics and differences.

Table 1 shows the essential statistical characteristics of the two-time series formed by the logarithmic returns of CSI 300 and S&P 500. It can be seen from the table that both time series exhibit negative skewness, −0.327415 and −0.771621 respectively, indicating that both time series have left-skewed characteristics; the logarithmic returns of S&P 500 and CSI 300 are both positively peaked and greater than 3, indicating that both have peaked and thick-tailed characteristics; the Jarque-Bera statistics of the two-time series are both significant, neither of which obeys the normal distribution, indicating that both time series have non-Gaussian characteristics.

**Table 1:** Descriptive statistical characteristics.

	CSI 300	S&P 500
Mean	−2.22E-05	0.000305
Median	3.95E-06	0.000851
Maximum	0.057774	0.089683
Minimum	−0.082087	−0.127652
Std. Dev.	0.012960	0.013897
Skewness	−0.327415	−0.771621
Kurtosis	5.953662	16.03408
Jarque-Bera	463.7481	8782.284
Probability	0.000000	0.000000

Table 2 is the unit root test result from the Prob. Column, it can be seen that the P-values of the ADF test of the CSI 300 and S&P 500 time series are both 0.0000 and the statistics are −35.21309 and −10.55667, respectively, which are much smaller than any significance level (1 %, 5 %, 10 %) critical value, which means that the null hypothesis is rejected. The alternative hypothesis is accepted; that is, it is considered that the CSI 300 and S&P 500 time series do not have unit roots and are stationary. This indicates that the two indices’ price and yield changes do not have apparent trends and periodicity, and their historical data cannot be used to predict future trends. This does not mean that the price changes of Chinese and American stock markets are completely random and irregular, but that the price changes of Chinese and American stock markets are influenced by many complex factors, which are difficult to quantify and capture, thus leading to the unpredictability of price changes. Table 1 has shown that the yield distributions of CSI 300 and S&P 500 are generally not distributed but peaked and negatively skewed distributions, which indicates that the data of Chinese and American stock market indices do not conform to the hypothesis of efficient market theory, are not entirely random walks, but have a certain degree of dependence and nonlinearity. The Chinese and American stock markets are more in line with the view of fractal market theory. The Chinese and American stock market price changes are fractal movements affected by investors' irrational behaviour and expectations. Therefore, research hypothesis 2 is established.

**Table 2:** Unit root test.

	Test statistic	Test critical values			Prob.
		1 %	5 %	10 %	
CSI 300	−35.21309	−3.965617	−3.413514	−3.128804	0.0000
S&P 500	−10.56667	−3.965667	−3.413539	−3.128819	0.0000

Table 3, the Pearson correlation coefficient between CSI 300 and S&P 500 is 0.032, and the significance level is 0.271, meaning there is no linear correlation between CSI 300 and S&P 500, or the linear correlation between CSI 300 and S&P 500 is extremely weak. The two should be a nonlinear correlation or a complex dynamic relationship.

**Table 3:** Pearson correlation test.

		CSI 300	S&P 500
CSI 300	Pearson Correlation	1	0.032
	Sig. (2-tailed)		0.271
	Sample	1216	1216
S&P 500	Pearson Correlation	0.032	1
	Sig. (2-tailed)	0.271	
	Sample	1216	1216

According to Table 4, the result of the Granger causality test of CSI 300 on S&P 500 is as follows: F statistic is 0.36716, P value is 0.6928. This means the null hypothesis cannot be rejected; CSI 300 has no Granger causal relationship with S&P 500. The result of the Granger causality test of S&P 500 on CSI 300 is as follows: F statistic is 3.85981, P value is 0.0213. This means the null hypothesis can be rejected; S&P 500 has a Granger causal relationship with CSI 300. It is comprehensively judged that when the lag order is 2, S&P 500 has a linear Granger causal relationship with CSI 300, while CSI300 does not have a linear Granger causal relationship with S&P 500. This shows that the historical information on the S&P 500 can help predict the future value of the CSI 300, while the historical information on the CSI 300 cannot help predict the future value of the S&P 500.

**Table 4:** Granger causality test, sample: 1 1216, lags: 2.

Null Hypothesis	Obs	F-Statistic	Prob.
S&P 500 does not Granger Cause CSI 300	1214	0.36716	0.6928
CSI 300 does not Granger Cause S&P 500		3.85981	0.0213

### 4.2 Characteristics analysis

#### 4.2.1 Cross-correlation test result analysis

**Fig. 2:**
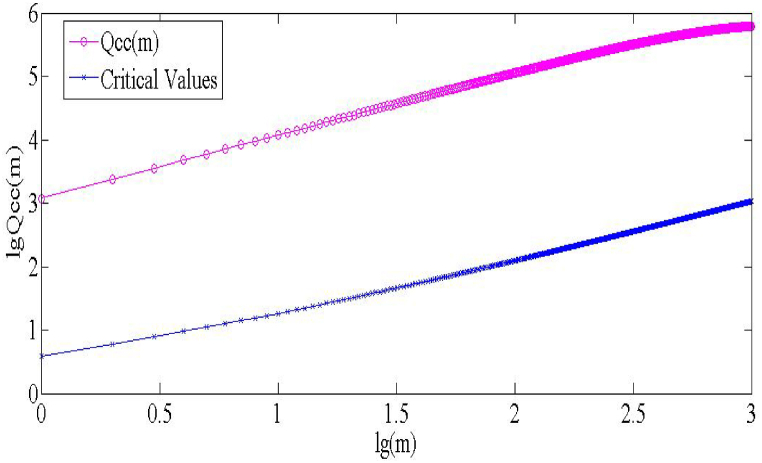
Cross-correlation test.

In Fig. 2, the pink line represents the cross-correlation statistic Qcc(m) of CSI 300 and S&P 500, and the blue line represents the critical value of the $\chi^{2}$ distribution. With degrees of freedom ranging from 1 to 1000, Qcc(m) is always more significant than the critical value of the chi-square distribution with a significance level of 5 % [31], indicating a significant long-term cross-correlation between CSI 300 and S&P 500. Fig. 2 shows that as the degree of freedom m increases, both Qcc(m) and the $\chi^{2}$ distribution show an increasing trend. However, the increase in Qcc(m) is more remarkable, indicating that the cross-correlation between CSI 300 and S&P 500 increases with the increase in the lag order; that is, the impact of the yield changes of CSI 300 and S&P 500 on each other has a longer duration. In general, there is a complex interaction mechanism between CSI 300 and S&P 500, which may involve multiple factors such as market sentiment, policy changes, globalization or de-globalization; this long-range cross-correlation between CSI 300 and S&P 500 may have a significant impact on investors' decision-making and risk management.

#### 4.2.2 DCCA correlation coefficient

Calculate the cross-correlation coefficient of CSI 300 and S&P 500 according to the size of different scales.

Table 5 shows that CSI 300 and S&P 500 show weak linear correlation at different scales. This is consistent with the result of the Pearson correlation test in Table 3, which confirms that the long-range cross-correlation between CSI 300 and S&P 500 should be nonlinear or a complex dynamic relationship, which may be affected by multiple nonlinear factors and complex mechanisms such as market efficiency, fractal characteristics, and chaotic phenomena. In general, research hypothesis 1 is established.

**Table 5:** Cross-correlation coefficient.

Scale	2	4	8	16	32	64	128	256	512
$\rho_{DCCA}$	0.0000	0.0000	0.0000	0.0001	0.0002	0.0004	0.0009	0.0015	0.0036
Intensity	very weak	very weak	very weak	very weak	very weak	very weak	weak	weak	weak

Note: In Table 5, when the scale is 2, 4, and 8, $\rho_{DCCA}$ = 0.0000 is due to the retention of 4 decimal places; if more decimal places are taken, $\rho_{DCCA}$ is not strictly zero.

#### 4.2.3 Multifractal characteristics analysis

Using the MF-DCCA model to calculate and analyze the logarithmic returns of CSI 300 and S&P 500, respectively, this paper takes the time scale as 4≤s≤N/4, where N is the length of the time series.

**Fig. 3:**
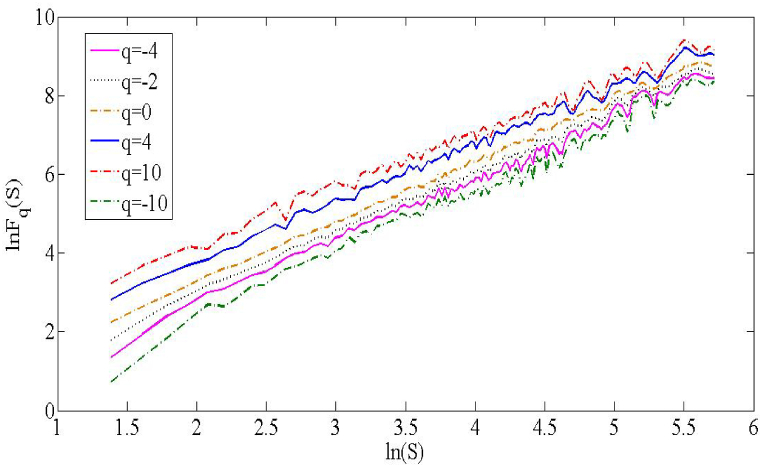
CSI 300和S&P 500 fluctuation function graph.

It can be seen from Fig. 3 that when q changes, the slope between $\ln\;F_{q}\left(s\right)$ and ln(s) also changes, indicating that there are multiple fractal characteristics between CSI 300 and S&P 500; that is, they exhibit different complexity and irregularity at different scales. Specifically, when q = 0, $\ln\;F_{q}\left(s\right)$ and ln(s) shows an approximate linear relationship, indicating that there is a long-term memory effect between CSI 300 and S&P 500; when q < 0, the slope between $\ln\;F_{q}\left(s\right)$ and ln(s) gradually increases, indicating that the small fluctuations between CSI 300 and S&P 500 have a weak correlation; when q > 0, the slope between $\ln\;F_{q}\left(s\right)$ and ln(s) gradually decreases, indicating that the large fluctuations between CSI 300 and S&P 500 have a strong correlation.

**Fig. 4:**
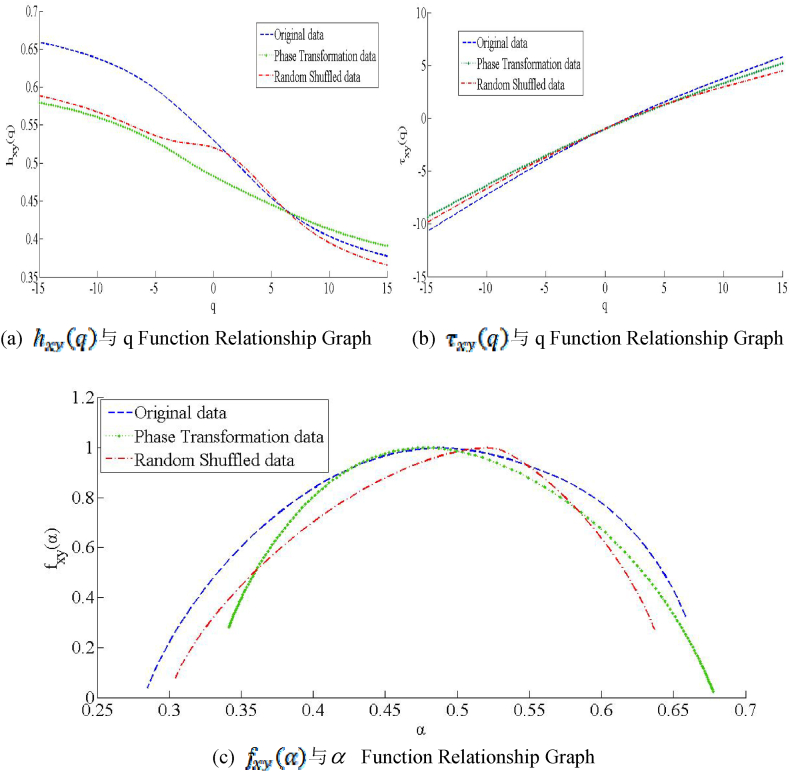
Multifractal characteristics graph.

Fig. 4(a), (b), and (c) illustrate the generalized Hurst index graph, the scaling index, and the multifractal spectrum of CSI 300 and S&P 500, respectively, based on the MF-DCCA model. Fig. 4(a) shows the generalized Hurst index graph of CSI 300 and S&P 500, demonstrating the multifractal characteristics of the indices. Fig. 4(b) presents the scaling index graph, which highlights the nonlinear behaviors under different market conditions. Fig. 4(c) displays the multifractal spectrum, illustrating the complexity and heterogeneity at different scales in the CSI 300 and S&P 500. These figures reveal that both the CSI 300 and S&P 500 exhibit significant multifractal characteristics, indicating that they possess distinct statistical properties across different scales, which reflects their high complexity and heterogeneity. The multifractal spectrum curves of CSI 300 and S&P 500 are relatively broad, indicating that they have strong singularity, which means that they both have high uncertainty and volatility and are difficult to predict with simple models; the curves of CSI 300 and S&P 500 are relatively high, indicating that they have large fractal dimensions, that is, the proportion of regions with the same singularity index is relatively large, which means that they have good robustness and self-organization ability, and can adapt to environmental changes.

#### 4.2.4 Causes of multifractal characteristics

In order to explore the source of multifractal characteristics, the original yield time series of CSI 300 and S&P 500 are randomly rearranged and phase processed. These two methods are usually used to test whether the multifractal characteristics of time series are affected by long-term memory effects or nonlinear effects [32]. The random rearrangement method shuffles the time series data, thus eliminating the long-term memory effect in the original series. The phase processing method randomly shuffles the phases after the Fourier transformation of the time series, thus preserving the long-term memory effect in the original series but eliminating the nonlinear effect. Suppose the multifractal characteristics of the time series data are weakened after random rearrangement and phase processing. In that case, it indicates that the long-term memory effect and nonlinear effect are the main reasons for the existence of multifractal characteristics in the original time series.

According to the results in Table 6, when q = 2, the Hurst index $h_{xy}\left(q\right)$ = 0.5055 > 0.5 of the original sequence, indicating that there is a long-range correlation between CSI 300 and S&P 500, that is, the price index fluctuation of one market will affect the price index of another market, reflecting the long-term memory effect between Chinese and American stock markets. It can be seen from Fig. 4(b) that the Renyi index $\tau_{xy}\left(q\right)$ of the original sequence is a strictly monotonic increasing convex function of q, and there is an apparent nonlinear relationship between $\tau_{xy}\left(q\right)$ and q, indicating that there is a nonlinear effect between CSI 300 and S&P 500, that is, they exhibit different dynamic behaviours in different situations. In order to further explore the reasons why CSI 300 and S&P 500 exhibit multifractal characteristics, multifractal analysis is performed on the original sequence, randomly shuffled sequence, and phase-processed sequence. It can be seen from Fig. 4 that the randomly shuffled sequence and the phase-processed sequence still exhibit multifractal characteristics. However, their Hurst index $h_{xy}\left(q\right)$, Renyi index $\tau_{xy}\left(q\right)$ and multifractal spectrum differ from the original sequences. Specifically, the Hurst index $h_{xy}\left(q\right)$ and Renyi index $\tau_{xy}\left(q\right)$ change less, and the width of the multifractal spectrum narrows, indicating that the long-term memory effect and nonlinear effect are the main reasons CSI 300 and S&P 500 exhibit multifractal characteristics. Therefore, research hypothesis 3 is established.

**Table 6:** Hurst index.

q-order	$h_{xy}\left(q\right)$		
	Original data	Phase transformation data	Random shuffled data
−10	0.6376	0.5603	0.5671
−8	0.6248	0.5494	0.5552
−6	0.6079	0.5359	0.5422
−4	0.5855	0.5195	0.5312
−2	0.5586	0.5003	0.5257
0	0.5301	0.4827	0.5201
2	0.5033	0.4673	0.4994
4	0.4683	0.4525	0.4737
6	0.4412	0.4383	0.4415
8	0.4199	0.4251	0.4150
10	0.4036	0.4132	0.3952
$\Delta h_{xy}\left(q\right)$	0.2340	0.1471	0.1719

According to Table 6, the Hurst index $h_{xy}\left(q\right)$ and the difference $\Delta h_{xy}\left(q\right)$ of the original time series, the randomly shuffled sequence, and the phase-processed sequence are compared. Additionally, Table 7 compares the multifractal spectrum width among the original time series, the randomly shuffled sequence, and the phase-processed sequence. The results indicate that these indicators decrease after random rearrangement and phase processing, suggesting that the multifractal characteristics are weakened. This finding implies that the long-term memory and nonlinear effect in the original time series are the primary reasons for the multifractal characteristics observed in the CSI 300 and S&P 500. Specifically, after random rearrangement, as shown in Table 6, the Hurst index $h_{xy}\left(q\right)$ value decreases from 0.5671 to 0.3952, and the difference $\Delta h_{xy}\left(q\right)$ changes from 0.2340 to 0.1719. Meanwhile, as shown in Table 7, the width of the multifractal spectrum decreases from 0.3741 to 0.3327. This shows that random rearrangement disrupts the long-term memory effect in the original time series, thereby weakening the multifractal characteristics between CSI 300 and S&P 500. After phase processing, as highlighted in Table 6, the Hurst index $h_{xy}\left(q\right)$ value decreases from 0.5603 to 0.4132, and the difference $\Delta h_{xy}\left(q\right)$ changes from 0.2340 to 0.1471. Similarly, as shown in Table 7, the width of the multifractal spectrum decreases from 0.3741 to 0.3362. This suggests that phase processing disrupts the nonlinear effect in the original time series, thus weakening the multifractal characteristics between the CSI 300 and S&P 500.

**Table 7:** Multifractal spectrum parameters.

	$\alpha_{\min}$	$\alpha_{\max}$	$\Delta \alpha_{xy}$	$\Delta f_{xy}\left(\alpha \right)$
Original data	0.2847	0.6588	0.3741	0.27620
Phase transformation data	0.3413	0.6775	0.3362	0.25732
Random shuffled data	0.3041	0.6368	0.3327	0.19674

## 5 Conclusion

This paper analyzes the cross-correlation between Chinese and American stock markets using the multifractal detrended cross-correlation analysis (MF-DCCA) method and explores its multifractal characteristics and influencing factors. This paper's main findings, main contributions, main implications, main limitations and future directions are discussed as follows:

The paper finds that the cross-correlation between Chinese and American stock markets exhibits multifractal characteristics, that is, different long-term memory effects, complexity and irregularity at different scales. The paper also finds that the multifractal characteristics are mainly caused by the long-term memory effect and nonlinear effect, that is, the historical changes and the dynamic behaviours of the two markets. The paper further finds that the data of the two markets do not support the efficient market theory but are more consistent with the fractal market theory.

The main contributions of this paper are:(1)This paper provides a new perspective and method for analyzing the cross-correlation between Chinese and American stock markets, which can capture the nonlinear and non-Gaussian features of the cross-correlation and reveal the multifractal characteristics and influencing factors of the cross-correlation. (2)This paper reveals the multifractal characteristics and influencing factors of the cross-correlation between Chinese and American stock markets, which are rarely studied in the existing literature and provide evidence for the fractal market theory.

This paper has specific implications and significance for investors and regulators in Chinese and American stock markets. Understanding the multifractal characteristics and influencing factors of the cross-correlation can help investors better grasp the market trend and risk and formulate reasonable investment strategies and decisions. Understanding the multifractal characteristics and influencing factors of the cross-correlation can help regulators better monitor and warn of abnormal market fluctuations and crises and formulate effective regulatory policies and measures.

This paper has shortcomings and limitations. For example, this paper only selects two stock indices as sample data. It does not consider other factors that may affect the two markets, such as macroeconomic indicators, political events, market sentiment, etc. This paper also needs to give an in-depth explanation and theoretical support for the multifractal characteristics of cross-correlation. However, it only describes and verifies them from the data analysis perspective. Therefore, future research can be expanded and deepened from the following aspects: (1)Expand the scope and quantity of sample data, including more stock indices or individual stocks and other variables that may affect the two markets, to improve the universality and representativeness of the research. (2)Introduce more multifractal analysis methods, such as the fluctuation function method, generalized dimension method, singular spectrum method, etc., to enhance the reliability and validity of the research. (3)Explore the mechanisms and causes behind the multifractal characteristics of the cross-correlation, such as investor behavior, information transmission, market efficiency, etc., to increase the research's theoretical significance and practical value.

## Data availability statement

The datasets/codes are available from the corresponding author upon reasonable request.

## Ethics declarations

Review and approval by an ethics committee was not needed for this study because it did not involve human subjects, the data used were publicly available, and no personally identifiable information was collected.

## CRediT authorship contribution statement

**Yijun Chen:** Conceptualization. **Jun-hao Zhang:** Formal analysis, Data curation. **Lei Lu:** Methodology, Formal analysis. **Zi-miao Xie:** Data curation.

## Declaration of competing interest

The authors declare the following financial interests/personal relationships which may be considered as potential competing interests: Yijun Chen reports administrative support was provided by 10.13039/501100003459Guizhou University of Commerce. If there are other authors, they declare that they have no known competing financial interests or personal relationships that could have appeared to influence the work reported in this paper.
